# Homework self-regulation strategies: a gender and educational-level invariance analysis

**DOI:** 10.1186/s41155-017-0062-z

**Published:** 2017-04-03

**Authors:** Irene Cadime, Joana Cruz, Carla Silva, Iolanda Ribeiro

**Affiliations:** 1 Centro de Investigação em Estudos da Criança (CIEC), Universidade do Minho, Braga, Portugal; 2 Centro de Investigação em Psicologia para o Desenvolvimento (CIPD), Universidade Lusíada - Norte, Porto, Portugal; 3 Escola de Psicologia, Universidade do Minho, Braga, Portugal

**Keywords:** Homework Behavior Questionnaire, Self-regulation, Measurement invariance, Gender differences, Elementary and middle school students

## Abstract

This study investigates the measurement invariance as a function of gender and educational level of the Homework Behavior Questionnaire (Ktpc), an instrument developed to assess students’ homework self-regulation strategies. A sample of 1400 elementary and middle school students was used. Results of confirmatory factor analysis indicated a good fit of the theoretical model composed of three dimensions: planning, execution and evaluation of the homework completion. The results also provided evidence for the existence of metric invariance and partial scalar measurement invariance across boys and girls and across the elementary school and the middle school students. The reliability of the scores in the three dimensions was high. Girls obtained higher scores than boys in planning, execution and evaluation. Middle school students had lower scores in planning compared to the elementary school students. These findings are discussed, and their implications for practice are highlighted.

## Background

Homework includes the set of prescribed tasks to students by teachers to be held outside school hours. Tutoring, preparation for tests and examinations, supervised study in school, correspondence study courses at home and extra-curricular activities such as sport, as well as study activities self-initiated by students, cannot be considered homework (Cooper, 2001). The positive effects of homework completion on students’ academic achievement across several subject matters have been demonstrated in a large number of studies (see, for example, the meta-analyses by Cooper, Robinson, & Patall, 2006, and Fan, Xu, Cai, He, & Fan, 2017).

Homework not only contributes to academic performance, at a general or specific level (i.e. math/science), but has also been associated with the students’ self-regulation abilities. The relationship between the two variables can be understood in the light of the demands that students must deal with when doing their homework. The accomplishment of homework includes a sequential set of three phases. The first comprises the prescription of the work done by the teacher and takes place in the classroom, the second takes place outside the classroom and consists of the execution of the prescribed tasks, and the last phase occurs upon return of the student to the classroom, after the work is done (Cooper, 1989, 2001; Coulter, 1979; Rademacher, 2000). In the second stage, students are responsible for doing the work prescribed by their teachers and managing time, spaces and environments, and even for seeking help whenever needed (Cooper, 2001; Corno, 2000; Trautwein & Koller, 2003). They should also accomplish the tasks in time, control the possible internal and external distractors, decide which aids they will use and check if all the prescribed tasks are complete (Epstein & Van Voorhis, 2001). Therefore, a successful homework completion demands self-regulation abilities, and some instruments that assess the self-regulatory components that are present during the execution of homework have been developed.

One of these instruments is the Homework Management Scale (Xu, 2008; Yang & Xu, 2015), which is a self-report measure for high school students composed of 22 items distributed by five subscales: arranging environment, managing time, handling distraction, monitoring motivation and controlling emotion. Studies with American (Xu, 2008) and Chinese (Yang & Xu, 2015) samples of 11th graders demonstrated adequate reliability. In both samples, confirmatory factor analysis results provided empirical evidence for the five-factor structure. Although invariance of this structure was demonstrated across calibration and validation samples (Xu, 2008), no invariance studies were conducted considering variables that play a role on self-regulation, such as gender. Studies focusing on gender differences in self-regulated learning found that girls are more self-regulated than boys (Xu & Corno, 2006), spend more time doing homework (Rosário, Mourão, Núñez, González-Pienda, & Valle, 2006), do a better behaviour regulation (Weis, Heikamp, & Trommsdorff, 2013) and take more initiative to manage their homework (Xu & Wu, 2013).

Another instrument that includes an assessment of the homework self-regulation components is the Self-Assessment Questionnaire: Homework (SAQ; Hong, Peng, & Rowell, 2009). The SAQ is composed of 34 items that measure homework utility value and intrinsic value (dimensions related with the task value), effort and persistence (dimensions related with the motivational outcome) and the planning and self-checking applied during the homework process (dimensions related with the metacognitive strategy use). Hong, Peng, and Rowell (2009) administered the SAQ to groups of 7th and 11th graders and found grade-level differences in the scores of the six SAQ dimensions, with the second group obtaining significantly lower scores than the first. They concluded, as a consequence, that “older Chinese students perceived homework as less useful, enjoyed doing homework less, expended less effort, persisted less, and engaged in planning and self-checking less than did younger students” (Hong et al., 2009, p. 274). In the same study, no gender differences were found. Nonetheless, no evidence for the grade and gender measurement invariance of the SAQ was provided, which is essential to guarantee the validity of these findings.

The Homework Distraction Scale (HDS; Xu, 2015) assesses one specific aspect of self-regulation in homework completion: the (in)ability to suppress distractors and maintain the attention in the homework task. The HDS is composed of six items that the students must rate using a Likert-type scale and that are organized into two dimensions: (a) conventional distraction (e.g. *Start conversations unrelated to what I’m doing*) and (b) tech-related distraction (e.g. *Stop math homework to play online games or video games*). Xu, Fan, and Du (2015) tested the two-factor structure and its measurement invariance across gender using a sample of 796 Chinese 8th graders. They found evidence for the existence of metric invariance between boys and girls. However, given that scalar invariance was not tested, no mean comparisons between both gender groups were performed.

As a summary, these instruments measure distinct self-regulation abilities during homework completion and have been developed to assess middle and high school students, probably mirroring the fact that most of the research on homework completion and its related variables is conducted at these stages (e.g. Iflazoglu & Hong, 2012; Lau, Kitsantas, & Miller, 2015; Lee, Lee, & Bong, 2014; Núñez et al., 2015; Regueiro, Suárez, Valle, Núñez, & Rosário, 2015; Valle et al., 2016; Xu, 2011; Xu & Wu, 2013; Yang & Xu, 2015). The development of scales that evaluate self-regulation of homework behaviours in younger children remains an important research issue. Moreover, all of the reviewed instruments are self-report measures and, as Xu (2008) indicates, “there is a need to incorporate other measures of homework behaviors over time (e.g., student’s homework behaviors as recorded and perceived by their teachers and their parents) to complement students’ self-reports” (p. 320) in order to have a more complete and reliable understanding of this issue.

The Homework Behavior Questionnaire (Ktpc) was developed to assess self-regulation abilities in homework completion but focus homework as a sequential process that involves self-regulatory skills. Homework models (Cooper, 2001; Corno, 2000; Coulter, 1979; Rademacher, 2000), as well as Zimmerman’s (2000) cyclical model of self-regulated learning, were used to guide the development of the Ktpc. The questionnaire is centred in the homework’s second phase, which corresponds to assignment execution, which usually occurs at home or in community contexts (Cooper, 2001; Coulter, 1979; Rademacher, 2000). Therefore, the Ktpc was developed in order to measure the processes, beliefs and behaviours that tend to occur during three steps: homework planning, execution and evaluation. Moreover, the questionnaire was developed to assess the behaviours of students from different educational levels—elementary school (grades 1–4) and the first cycle of middle school (grades 5–6)^1^—based on the information provided by the students’ parents or other tutors. Given that the factor structure of the Ktpc was not previously tested using confirmatory factor analysis, the first two goals of this study were to test the fit of the theoretical model to the data and to investigate the reliability of the scores obtained in the Ktpc.

As was previously referred, research has found consistent differences between girls and boys in the self-regulation abilities. Therefore, it is crucial to check the measurement invariance of any instrument that focuses on these abilities so that meaningful comparisons can be performed between male and female students. In the studies of the previous referred instruments that measure homework self-regulation components (Hong et al., 2009; Xu, 2008; Xu, 2015; Yang & Xu, 2015), this was not examined. Similarly, given that research found that older students are less self-regulated during homework completion (Hong et al., 2009) and that Ktpc was developed to assess children who attend two different educational levels, measurement invariance across these levels must also be guaranteed. Therefore, the third goal of this study was to investigate the measurement invariance of the Ktpc as a function of gender and educational level. Only after guaranteeing measurement invariance, meaningful group comparisons using the Ktpc can be performed.

Hence, the research questions of this study were as follows: (a) Does a multidimensional structure composed of three factors—planning, execution and evaluation—fit the data obtained in the Ktpc? (b) Are the scores of the Ktpc reliable? and (c) Is the factor structure invariant between boys and girls and between students from elementary and middle schools?

## Methods

### Participants and procedure

The sample was recruited by convenience, using a snowballing sampling technique for data collection. Formal authorizations from the board of the schools were collected prior to the questionnaire administration. The boards of seven public schools, located in the district of Porto (Portugal), were contacted and agreed to participate. These schools had a total of 1014 students from grades 1–4 and 611 students from grades 5–6. An informed consent form was distributed to the parents of these students informing them of the objectives of the study and asking for their collaboration, along with the questionnaires. This procedure was performed with the collaboration of the Psychology Services of the schools in which the study was conducted. The questionnaires were answered at home by the parents. The questionnaires were anonymous and were delivered and returned using closed envelops. The response rate was 86.15%. Therefore, the information regarding the behaviour during homework of 1400 students from Portuguese elementary (grades 1 to 4) and middle (grades 5 to 6) schools was collected. Table 1 displays the number of students in each grade and by gender. The students were equally distributed by the six grades, and the number of boys and girls was equivalent in all grade levels, *χ* ^2^(5) = 9.046, *p* = .107. However, given that the number of grade levels in elementary school was higher, the number of students from the middle school grades was substantially lower (*n* = 557) than the number of students from the elementary school grades (*n* = 841).

**Table 1:** Distribution of students according to grade and gender

	Gender			Age (years)
Grade	Boys	Girls	No information	*M* (SD)
1	109 (15.5%)	103 (15.1%)	5 (38.5%)	6.47 (0.519)
2	105 (14.9%)	112 (16.4%)	1 (7.7%)	7.52 (0.528)
3	89 (12.7%)	106 (15.5%)	1 (7.7%)	8.54 (0.539)
4	95 (13.5%)	113 (16.5%)	2 (15.4%)	9.55 (0.628)
5	146 (20.8%)	114 (16.7%)	1 (7.7%)	10.53 (0.592)
6	159 (22.6%)	135 (19.7%)	2 (15.4%)	11.61 (0.724)
No information	0 (0%)	1 (0.1%)	1 (7.7%)	–

*M* mean, *SD* standard deviation

### Measures

The Ktpc is composed of three subscales: planning (six items), execution (seven items) and evaluation (eight items). The first subscale includes goal-setting/planning and is related to self-management or structuring either one’s self-processes (i.e. behaviours, thoughts, emotions) or the social environment, before engaging in homework assignments. The second consists of behaviours that tend to emerge during homework execution, namely those related to self-reinforcement, persistence and seeking support. The third includes behaviours that tend to emerge after the assignments are finished, such as self-evaluation, self-correction, homework revision and seeking homework feedback.

Each item consists of a statement, and the parents or tutors must rate the frequency of specific children’s homework behaviours, using a 5-point Likert scale response format (0 = never, 1 = rarely, 2 = sometimes, 3 = often, 4 = always). The items are presented in the Appendix.

### Statistical analyses

Analyses were conducted with Mplus, version 7 (Muthén & Muthén, 2012). Because the distribution of the variables was non-normal, the maximum likelihood estimation with robust standard errors (MLR) was used. Confirmatory factor analysis (CFA) was used to test the fit of the three-factor model in each of the four groups: male students, female students, elementary school students and middle school students. To assess the global fit of the tested models, the following criteria were used: the chi-square (*χ* ^2^) values, the ratio between the chi-square and the degrees of freedom (*χ* ^2^/df), the comparative fit index (CFI), the root mean square error of approximation (RMSEA) and the standardized root mean square residual (SRMR). Model fit was considered acceptable when *χ* ^2^/df was lower than 3.00, CFI values were higher than .90, RMSEA lower than .08 and SRMR lower than .10 (Schermelleh-Engel, Moosbrugger, & Müller, 2003). Composite reliability was calculated for each factor and values higher than .70 were considered adequate (George & Mallery, 2002; Hair, Black, Babin, & Anderson, 2009).

After fitting the model separately, in a second step, multi-group CFA was performed to test the invariance of the structure across genders and educational levels, following the guidelines indicated by van de Schoot, Lugtig, and Hox (2012) and Byrne (2012). First, a configural model (model 0), where loadings and intercepts were freely estimated, was tested. In model 1, metric invariance was tested, where the factor loadings were constrained but the intercepts were freely estimated. In model 2, scalar invariance was tested, where both loadings and intercepts were constrained to be equal across both samples. Evidence for the invariance of the model across both samples is achieved when the constraint of parameters performed in testing the subsequent models does not worsen the fit indices. To perform this comparison, the Satorra–Bentler scaled chi-square difference test was calculated (ΔSB − *χ* ^2^). The comparison index Bayesian information criteria (BIC) was also used: the model with the lowest value was considered to be the one that best represents the data. Moreover, two additional criteria were considered, as recommended by Cheung and Rensvold (2002) and Chen (2007): (a) the difference in CFI (ΔCFI) that should be equal or lower than .01 and (2) the difference in RMSEA (ΔRMSEA) that should be equal or lower than .015. When full scalar invariance was not achieved, partial invariance was established by estimating freely the parameters identified after examining the Lagrange multiplier tests. After establishing the invariance of the factor structure, differences in the latent means between the gender groups and the educational-level groups were calculated.

## Results

**Fig. 1:**
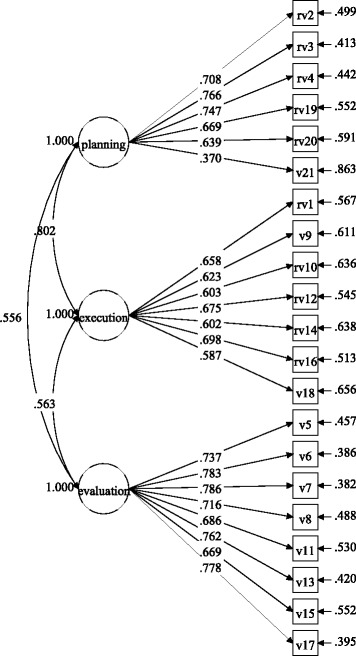
Factor loadings for the three-factor model in the girls’ group

**Fig. 2:**
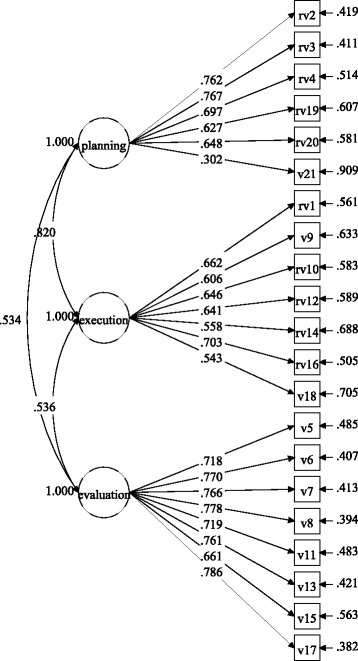
Factor loadings for the three-factor model in the boys’ group

**Fig. 3:**
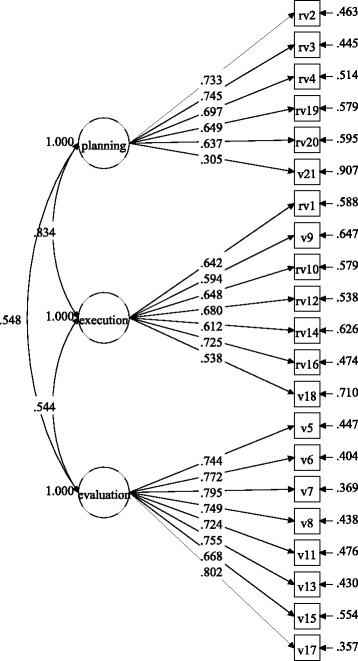
Factor loadings for the three-factor model in the elementary school group

**Fig. 4:**
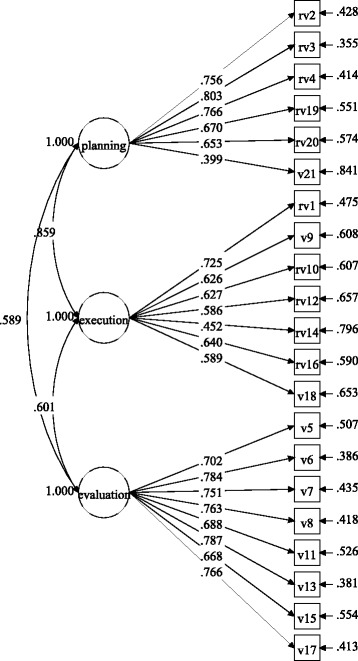
Factor loadings for the three-factor model in the middle school group

Table 2 presents the model fit for each gender and educational-level group. The three-factor model had an acceptable fit in all gender and educational-level groups, as indicated by the CFI, RMSEA and SRMR values, although the *χ* ^2^/df slightly exceeded the reference values. Figures 1 and 2 show the factor loadings for the items in each gender group, and Figs. 3 and 4 display the factor loadings in each educational level. As can be seen in Figs. 1, 2, 3 and 4, all factor loadings were higher than .30.

**Table 2:** Model fit for the three-factor model by gender and educational level

Group	*χ* ^2^	df	*P*	*χ* ^2^/df	CFI	RMSEA	90% CI RMSEA	SRMR	BIC
Girls	643.68	186	<.001	3.46	.91	.06	.06–.07	.06	38975.58
Boys	735.50	186	<.001	3.95	.90	.07	.06–.07	.06	41200.49
Elementary school	753.33	186	<.001	4.05	.91	.06	.06–.07	.05	48435.61
Middle school	639.44	186	<.001	3.44	.90	.07	.06–.07	.07	32229.21

*CFI* comparative fit index, *RMSEA* root mean square error of approximation, *SRMR* standardized root mean square residual, *BIC* Bayesian information criterion

### Reliability

Table 3 presents descriptive statistics and the reliability testing results as a function of gender and educational levels. Composite reliability values were higher than .80 for all subscales in all four groups and were particularly high for the evaluation subscale.

**Table 3:** Descriptive statistics and reliability coefficients

Dimension	Items	Girls		Boys		Elementary school		Middle school	
		*M* (SD)	CR	*M* (SD)	CR	*M* (SD)	CR	*M* (SD)	CR
Planning	6	24.403 (4.646)	.819	22.324 (5.077)	.808	23.701 (4.725)	.802	22.826 (5.301)	.838
Execution	7	25.999 (5.397)	.826	25.261 (5.546)	.817	24.861 (5.476)	.826	26.729 (5.307)	.804
Evaluation	8	27.647 (7.547)	.907	25.168 (7.662)	.909	26.278 (7.823)	.912	26.556 (7.519)	.906

*M* mean, *SD* standard deviation, *CR* composite reliability

### Gender invariance

Table 4 shows the results of the invariance testing across gender and educational levels. Regarding the gender invariance testing, the results for the configural model (model 0) indicated an acceptable fit, although the *χ* ^2^/df was out of the cut-off value of 3.00. The metric invariance model (model 1) fitted equally well, given that the ΔSB − *χ* ^2^ was non-significant, and the CFI and RMSEA differences did not exceed the reference values. The chi-square test of differences indicated that the scalar invariance model testing for boys and girls (model 2) fitted worse than model 1. Although the ΔRMSEA was lower than .015, the ΔCFI exceeded .01. The modification indices flagged the intercept of item 14, suggesting that it was not invariant. As a result, another model (model 3) was run to test partial scalar invariance. In this model, the identified intercept was unconstrained. Although the ΔSB − χ^2^ was significant, the ΔCFI and ΔRMSEA were lower than the reference values (see Table 4). Moreover, model 3 had the lowest BIC of all models. Taken together, these results indicate that model 3 did not fit worse than model 1, and provide evidence for the existence of partial strong factorial invariance across boys and girls. The comparison of the latent means indicated that girls obtained higher scores than boys in all three dimensions: planning (ΔM = .45, *p* < .001), execution (ΔM = .18, *p* < .01) and evaluation (ΔM = .34, *p* < .001). The results were similar for the execution dimension if item 14 was excluded in the comparison of latent means (ΔM = .20, *p* < .01).

**Table 4:** Measurement invariance across genders (*N* = 1387) and educational levels (*N* = 1398)

Model	*χ* ^2^	df	*p*	*χ* ^2^/df	CFI	RMSEA	90% CI RMSEA	SRMR	BIC	ΔSB − *χ* ^2^ (df)	ΔCFI	ΔRMSEA
Gender												
Model 0: configural	1378.467	372	<.001	3.706	.903	.062	.059–.066	.059	80267.581	–	–	–
Model 1: metric	1408.690	393	<.001	3.584	.902	.061	.058–.065	.063	80141.507	24.302 (21)	.001	.001
Model 2: scalar	1545.190	414	<.001	3.732	.891	.063	.059–.066	.077	80138.773	147.374 (21)^***^	.011	.002
Model 3^a^: partial scalar	1528.940	413	<.001	3.702	.892	.062	.059–.066	.077	80127.547	128.712 (20)^***^	.010	.001
Educational level												
Model 0: configural	1392.982	372	<.001	3.745	.904	.063	.059–.066	.059	80759.100			
Model 1: metric	1451.976	393	<.001	3.695	.901	.062	.059–.066	.068	80666.025	56.208 (21)^***^	.003	.001
Model 2: scalar	1749.108	414	<.001	4.225	.875	.068	.065–.071	.080	80851.854	339.662 (21)^***^	.026	.006
Model 3^b^: partial scalar	1537.974	410	<.001	3.751	.894	.063	.059–.066	.071	80632.473	90.717 (17)^***^	.007	.001

*CFI* comparative fit index, *RMSEA* root mean square error of approximation, *SRMR* standardized root mean square residual, *BIC* Bayesian information criterion, *ΔSB − χ* ^*2*^ Satorra–Bentler scaled chi-square difference test

***p < .001

^a^Estimating freely the intercept of item 14 in both samples

^b^Estimating freely the intercepts of items 12, 14, 16 and 18 in both samples

### Educational-level invariance

Regarding the educational-level invariance testing, the results for the configural model (model 0) indicated an acceptable fit, with all indices (excepting *χ* ^2^/df) being within the reference values. The chi-square test of differences indicated that the metric invariance model (model 1) fitted worse than the configural model. However, the ΔCFI and ΔRMSEA tests did not exceed the reference values and model 1 had a lower BIC than model 0, supporting the invariance of the factor loadings across students from both educational levels. The results for model 2 that tested scalar invariance indicated a poorer fit compared to the previous model: the chi-square test of differences was significant, the BIC value was higher than the one obtained for model 1 and the ΔCFI was higher than .01. The inspection of the modification indices led us to identify four intercepts (intercepts of items 12, 14, 16 and 18) that could improve the fit of the model if released, suggesting that these were not invariant. Consequently, a fourth model was run to test partial scalar invariance, where loadings and intercepts were constrained, excepting the four intercepts that were identified as non-invariant. Releasing these intercepts led to an improvement in the model fit (see Table 4). Therefore, partial measurement invariance across the elementary school and the middle school sample was established, and differences in the latent means between both groups were subsequently computed. When compared with the elementary school students, middle school students had lower results in planning (ΔM = −.17, *p* < .01). However, no differences between elementary and middle school students were found in execution (ΔM = .01, *p* = .92) and evaluation (ΔM = .04, *p* = .53). If the four items that had non-invariant intercepts were excluded (all from the execution dimension) in the comparison of latent means, the results were similar, as no differences between elementary and middle school students were found in homework execution strategies (ΔM = −.01, *p* = .93).

## Discussion

The Ktpc is an instrument constructed to assess students’ homework behaviour, as reported by parents or other tutors. Homework behaviour is related with what students do when dealing with homework, how they approach their work and how they manage their personal resources and homework settings (Valle et al., 2015). The first goal of this study was to test the fit of a model composed of three factors—planning, execution and evaluation—to the data obtained with the Ktpc in four groups: boys, girls, students from elementary school and students from middle school. The results from confirmatory factor analysis offer support to the validity of the instrument, with an acceptable fit of the proposed theoretical model to the data in all four groups. The three dimensions of the model are theoretically defined as essential self-regulated processes in homework execution: homework planning, homework execution and homework evaluation. In essence, the Ktpc provides information about how frequently an individual uses self-regulated strategies to complete assignments. Thus, students who score high on this scale will often regulate their behaviours, thoughts and emotions to finish homework and to associate the execution of the assignments to school outcomes.

The second goal was to explore the reliability of the results obtained in the Ktpc. Composite reliability results indicate that the scores in the Ktpc are highly reliable.

The third goal of this study was to investigate the measurement invariance of the three-dimensional structure between boys and girls and between students from elementary and middle schools. The results of this study indicated that the three-dimensional structure is partially invariant between boys and girls and between students from elementary and middle schools, thus allowing meaningful comparisons across groups. Gender differences were found in all three self-regulation dimensions of the homework, and these differences were favourable to girls. These findings are consistent with the bulk of research which indicates that not only are girls usually more self-regulated but they also invest more time and effort and apply better self-regulation strategies in completing homework (Rosário et al., 2006; Weis et al., 2013; Xu & Corno, 2006; Xu & Wu, 2013). However, these are contrary to the findings of Hong et al. (2009) which found no gender differences in homework planning and self-checking as measured by the SAQ. The differences in the cultural settings (Portugal versus China) and the differences in the educational levels assessed can explain the divergence between the results of the present study and the ones obtained in the study by Hong et al. (2009).

Differences were also found between elementary and middle school students, but only in homework planning. Elementary school students had higher results in planning when compared with middle school students. This result may reflect the progressive decreasing in the involvement in the studying activities and in the positive attitudes towards homework as the students advance in the educational system (Rosário et al., 2005). Hong et al. (2009) had also found that students from the 11th grade planned homework less than the 7th grade students. Taken together, these and our results can indicate that homework planning decreases as the school grade increases and that this decrease starts at early stages, but this hypothesis must be investigated using a longitudinal design in the future.

Although the differences in the groups’ latent means were similar, whether the non-invariant items were considered or excluded, future gender and educational-level comparisons of the scores obtained in the Ktpc should be interpreted cautiously, given that only partial measurement invariance was obtained.

All the items with non-invariant intercepts belonged to the execution subscale and had in common the fact that they are all related with the necessary self-control and ability to manage homework execution without the help of others, such as an adult (see the Appendix). Although research has shown that students tend to become gradually more autonomous in homework completion as they grow older, it has also indicated that students with higher academic achievement search more the adult supervision than students with lower academic achievement (for a review, see Corno & Mandinach, 2004). Additionally, the benefits of family help during homework completion are unclear. The results of some studies (e.g. Xu, 2007; Xu, Du, & Fan, 2016) indicated that family help was positively associated with the development of homework management abilities, but other studies (e.g. Silinskas, Kiuru, Aunola, Lerkkanen, & Nurmi, 2015) showed that excessive help, especially when children are perceived by their mothers as not very autonomous, led to poorer academic performance. Consequently, future studies should explore the pertinence of maintaining these items in the Ktpc.

## Conclusions

Although literature on self-regulation and homework is growing, fewer studies focus specifically on the metric properties of questionnaires of homework self-regulation behaviours, and as far as we know, no instruments have been developed to assess these behaviours in children from the initial school grades. The Ktpc is a step taken towards that goal and presents itself as a promising and reliable measuring instrument focused on the process of homework completion. This scale may also help teachers to develop interventions to foster self-regulated learning through homework improvement. Future research should focus on gathering other types of validity evidence for the Ktpc, such as the one based on the relationship to other variables. It would be particularly important to study the relationship of the scores in homework planning, execution and self-evaluation with the academic achievement across different school subjects and specific subject matters.

## Appendix

**Table 5:** Homework Behavior Questionnaire (Ktpc)

Item (in Portuguese)	English translation	Dimension
1. Quando sente dificuldades a realizar o trabalho de casa, desiste de os fazer.*	When he/she feels difficulties in performing homework, he/she gives up the task.*	Execution
2. Demora muito tempo a iniciar a realização do trabalho de casa.*	It takes him/her a long time to initiate homework.*	Planning
3. Cria desculpas para não fazer o trabalho de casa.*	He/she makes up excuses to avoid homework.*	Planning
4. Tem de ser lembrado/a para fazer o trabalho de casa.*	He/she only performs homework if someone reminds him/her.*	Planning
5. Quando acaba de fazer os trabalhos de casa avalia o seu desempenho, ou seja, aquilo que fez.	When he/she finishes homework, he/she evaluates what he/she did.	Evaluation
6. Quando acaba de fazer os trabalhos de casa avalia a eficácia do seu planeamento, ou seja, se planeou bem o que tinha de fazer.	When he/she finishes homework, he/she evaluates if his/her work was adequately planned.	Evaluation
7. Quando acaba de fazer os trabalhos de casa avalia a sua qualidade.	When he/she finishes homework, he/she evaluates the quality of the final result.	Evaluation
8. Quando acaba de fazer os trabalhos de casa relê as respostas.	When he/she finishes homework, he/she reads his/her answers again.	Evaluation
9. Parece confiar nas suas capacidades para resolver os trabalhos de casa.	He/she trusts his/her abilities to perform homework.	Execution
10. Costuma ficar frustrado/a e angustiado/a com os trabalhos de casa.*	He/she usually gets angry and frustrated with the homework. *	Execution
11. Quando acaba de fazer os trabalhos de casa revê o que fez e verifica se completou todas as tarefas.	When he/she finishes homework, he/she re-examines what he/she did and check if all tasks were completed.	Evaluation
12. Precisa de ajuda para fazer o trabalho de casa.*	He/she always needs help to perform homework.*	Execution
13. Quando acaba de fazer os trabalhos de casa revê a apresentação das respostas no caderno.	When he/she finishes homework, he/she re-examines the answers presentation in his/her workbook.	Evaluation
14. À mínima dificuldade sentida durante a realização do trabalho de casa pede logo ajuda.*	He/she always asks immediately for help when he/she feels even a little difficulty in homework.*	Execution
15. Quando acaba de fazer os trabalhos de casa faz uma previsão das respostas correctas.	When he/she finishes homework, he/she tries to guess how many correct answers he/she wrote.	Evaluation
16. Pede sempre ajuda quando faz o trabalho de casa, para acabar mais depressa.*	He/she always asks for helpwhen doing homework, so that he/she can finish as soon as possible.*	Execution
17. Quando acaba de fazer os trabalhos de casa revê a forma como o trabalho de casa está organizado.	When he/she finishes homework, he/she checks if the information is well organized.	Evaluation
18. Quando tem dificuldades no trabalho de casa tenta ultrapassá-las antes de pedir ajuda a algum adulto.	When he/she experiences difficulties in completing homework, he/she tries to overcome them before asking an adult for help.	Execution
19. Quando tem muito tempo para fazer os trabalhos de casa só os faz mesmo no final.*	When he/she has much time to complete homework, he/she only do it near the deadline.*	Planning
20. Antes de começar a fazer os trabalhos de casa, fica indeciso/a quanto às tarefas que deve fazer.*	Before he/she starts homework, he/she has doubts about which tasks should be done.*	Planning
21. Quando se prepara para fazer os trabalhos de casa, procura arranjar espaço suficiente para trabalhar.	When he/she prepares himself/herself to perform homework, he/she tries to find a good space to work.	Planning

*Note*: Items with an asterisk (*) should be reverse coded

## Footnotes

1 In Portugal, elementary school, also known as the first cycle of basic education, comprises grades 1 to 4 (children aged between 6 and 10 years old). Middle school comprises the second cycle of basic education (grades 5 to 6; children aged between 10 and 12 years old) and the third cycle of basic education (grades 7 to 9; children aged between 12 and 15 years old).

## References

[CR1] Byrne BM (2012). Structural equation modeling with Mplus: Basic concepts, applications
and programming.

[CR2] Chen FF (2007). Sensitivity of goodness of fit indexes to lack of
measurement invariance. Structural Equation Modeling: A Multidisciplinary
Journal.

[CR3] Cheung GW, Rensvold RB (2002). Evaluating goodness-of-fit indexes for testing
measurement invariance. Structural Equation Modeling: A Multidisciplinary
Journal.

[CR4] Cooper H (2001). The battle over homework: Common ground for administrators, teachers,
and parents.

[CR5] Cooper H (1989). Homework.

[CR6] Cooper H, Robinson J, Patall E (2006). Does homework improve academic achievement? A
synthesis of research, 1987–2003. Review of Educational Research.

[CR7] Corno L (2000). Looking at homework differently. The Elementary School Journal.

[CR8] Corno L, Mandinach EB, McInerney D, Van Etten S (2004). What we have learned about student engagement in the
past twenty years. Big Theories Revisited.

[CR9] Coulter F (1979). Homework: A neglected area of research. British Educational Research Journal.

[CR10] Epstein JL, Van Voorhis FL (2001). More than minutes: Teachers’ roles in designing
homework. Educational Psychologist.

[CR11] Fan H, Xu J, Cai Z, He J, Fan X (2017). Homework and students’ achievement in math and
science: A 30-year meta-analysis, 1986–2015. Educational Research Review.

[CR12] George D, Mallery P (2002). SPSS for Windows step by step: A simple guide and reference.

[CR13] Hair JF, Black WC, Babin BJ, Anderson RE (2009). Multivariate data analysis.

[CR14] Hong E, Peng Y, Rowell LL (2009). Homework self-regulation: Grade, gender, and
achievement-level differences. Learning and Individual Differences.

[CR15] Iflazoglu A, Hong E (2012). Relationships of homework motivation and preferences
to homework achievement and attitudes in Turkish students. Journal of Research in Childhood Education.

[CR16] Lau C, Kitsantas A, Miller A (2015). Using microanalysis to examine how elementary students
self-regulate in math: A case study. Procedia - Social and Behavioral Sciences.

[CR17] Lee W, Lee M, Bong M (2014). Testing interest and self-efficacy as predictors of
academic self-regulation and achievement. Contemporary Educational Psychology.

[CR18] Muthén LK, Muthén BO (2012). Mplus user’s guide.

[CR19] Núñez J, Suárez N, Cerezo R, González-Pienda J, Rosário P, Mourão R, Valle A (2015). Homework and academic achievement across Spanish
compulsory education. Educational Psychology.

[CR20] Rademacher JA (2000). Involving students in assignment
evaluation. Intervention in School and Clinic.

[CR21] Regueiro B, Suárez N, Valle A, Núñez JC, Rosário P (2015). La motivación e implicación en los deberes escolares a
lo largo de la escolaridad obligatoria. Revista de Psicodidáctica.

[CR22] Rosário P, Mourão R, Núñez J, González-Pienda J, Valle A (2006). SRL and EFL homework: Gender and grade
effects. Academic Exchange Quarterly.

[CR23] Rosário P, Mourão R, Soares S, Chaleta E, Gracio L, Núñez J, González-Pineda J (2005). Trabalhos de casa, tarefas escolares, auto-regulação e
envolvimento parental. Psicologia em Estudo.

[CR24] Schermelleh-Engel K, Moosbrugger H, Müller H (2003). Evaluating the fit of structural equation models:
Tests of significance and descriptive goodness-of-fit measures. Methods of Psychological Research Online.

[CR25] Silinskas G, Kiuru N, Aunola K, Lerkkanen M-K, Nurmi J-E (2015). The developmental dynamics of children’s academic
performance and mothers’ homework-related affect and practices. Developmental Psychology.

[CR26] Trautwein U, Koller O (2003). The relationship between homework and
achievement—still much of a mystery. Educational Psychology Review.

[CR27] Valle A, Pan I, Regueiro B, Suárez N, Tuero E, Nunes AR (2015). Predicting approach to homework in Primary school
students. Psicothema.

[CR28] Valle A, Regueiro B, Núñez J, Rodriguez S, Piñeiro I, Rosário P (2016). Academic goals, student homework engagement, and
academic achievement in elementary school. Frontiers in Psychology.

[CR29] van de Schoot R, Lugtig P, Hox J (2012). A checklist for testing measurement
invariance. European Journal of Developmental Psychology.

[CR30] Weis M, Heikamp T, Trommsdorff G (2013). Gender differences in school achievement: The role of
self-regulation. Frontiers in Psychology.

[CR31] Xu J, Fan X, Du J (2015). Homework management scale: Confirming the factor
structure with middle school students in China. Psychology in the Schools.

[CR32] Xu J (2007). Middle-school homework management: More than just
gender and family involvement. Educational Psychology.

[CR33] Xu J (2008). Validation of scores on the homework management scale
for middle school students. The Elementary School Journal.

[CR34] Xu J (2011). Homework purpose scale for middle school students: A
validation study. Middle Grades Research Journal.

[CR35] Xu J (2015). Investigating factors that influence conventional
distraction and tech-related distraction in math homework. Computers & Education.

[CR36] Xu J, Corno L (2006). Gender, family help, and homework management reported
by rural middle school students. Journal of Research in Rural Education.

[CR37] Xu, J, Du, J, & Fan, X. (2016). Self-regulation of mathematics homework behavior: An empirical investigation. *The Journal of Educational Research*. 10.1080/00220671.2015.1125837.

[CR38] Xu J, Wu H (2013). Self-regulation of homework behaviour: Homework
management at the secondary school level. The Journal of Educational Research.

[CR39] Yang F, Xu J (2015). Examining the psychometric properties of the Homework
Management Scale for high school students in China. Journal of Psychoeducational Assessment.

[CR40] Zimmerman BJ, Boekaerts M, Pintrich PR, Zeidner M (2000). Attaining self-regulation: A social cognitive
perspective. Handbook of self-regulation.

